# Exploring the gastric cancer care pathway in South Africa

**DOI:** 10.4102/phcfm.v17i1.4774

**Published:** 2025-04-30

**Authors:** Anishka Ramadhar, Juliana Kagura, Mazvita Muchengeti, Cameron Gaskill, Natasha Khamisa

**Affiliations:** 1 Division of Epidemiology and Biostatistics, School of Public Health, Faculty of Health Sciences, University of the Witwatersrand, Johannesburg, South Africa; 2 National Cancer Registry, National Health Laboratory Service, Johannesburg, South Africa; 3 DSI-NRF South African Centre of Excellence in Epidemiological Modelling and Analysis, Stellenbosch University, Stellenbosch, South Africa; 4 Department of Surgery, Faculty of Medicine, University of California San Francisco, San Francisco, United States; 5 Division of Health and Society, School of Public Health, Faculty of Health Sciences, University of the Witwatersrand, Johannesburg, South Africa

**Keywords:** gastric cancer, South Africa, care pathway, mapping, multi-disciplinary team, healthcare sector, diagnosis, treatment, management

## Abstract

**Background:**

Gastric cancer (GC) diagnosis and care data in South Africa (SA) is sparse, and SA has a high GC mortality rate. Mapping the GC care pathway is needed to explore its efficacy in association with the SA GC burden and mortality.

**Aim:**

The study aims to map the GC care pathway in SA from diagnosis to management by healthcare professionals (HCPs) involved in the GC patient journey and explore barriers and facilitators to the effective flow of the GC care pathway.

**Setting:**

Interviews conducted with South African HCPs were the data source used in this article for analysis. General physicians (GP) were the first contact point with chain-referral sampling sourcing other clinicians.

**Methods:**

Interviews were conducted via Microsoft Teams (MS Teams) and Google Meet with qualitative analyses via MAXQDA.

**Results:**

Themes identified were GC care pathway processes, public versus private healthcare system differences and care pathway challenges. Multidisciplinary team (MDT) care is practised for GC in SA, starting with the GP or nurse followed by gastroenterologist (GI), surgeon and pathologist. Thereafter, nurses, dieticians and palliative care specialists are involved. Healthcare sector differences are diagnosis time, GC staging, HCP and treatment access. Challenges include low GC index of suspicion by primary care clinicians (PCC) and *Helicobacter pylori (H. pylori)* detection.

**Conclusion:**

A MDT approach for optimal treatment and patient care may be the best method for prolonged life.

**Contribution:**

A South African national consensus for GC care via a MDT, emphasising early diagnosis to aid in a robust treatment plan for improved patient outcomes is warranted.

## Introduction

Gastric cancer (GC) is a substantial public health problem worldwide. In 2022, it was reported that GC was the 5th most common cancer and 5th leading cause of cancer-related mortality globally.^1^ The 5-year survival rate for GC is estimated to be below 20%, with males having double the frequency of GC than females.^2,3^ Gastric cancer incidence varies vastly between countries and regions, and this may be because of many factors such as infectious agents, genetics, environmental toxins and diet.^3^ Over 50% of new GC cases are identified in developing countries compared to developed countries.^3^

The 2022 South African National Cancer registry (NCR) report indicated that in South Africa (SA), GC makes up 1.92% of all cancers in males and 1.13% of all cancers in females,^4^ ranking as the 10th (804 cases) and 14th (518 cases) most common malignancy in males and females, respectively. The latest Statistics SA report (2018) shows GC as the 7th and 10th most common cause of cancer deaths in South African males and females, respectively.^5^ Delayed time to diagnosis results in many patients being diagnosed in the late stages of GC resulting in limited treatment options.^6^ Risk factors for GC in SA include a high salt diet, excessive alcohol and red meat consumption, *Helicobacter pylori (H. pylori)* infection, tobacco use, gastrointestinal reflux disease, obesity and a family history of GC.^7^

Care pathways for various conditions are used to strategise and implement a standardised patient centric care approach from diagnosis to post treatment phases.^8^ The goals of care pathways are clear communication between healthcare professionals (HCPs) and patients, thorough diagnostic workup and synchronisation with all members of the care team for optimal treatment tailored to each patient.^9^ Effective care pathways facilitate standardised flow of care processes while also keeping the individual patient in focus. This enables the pathway to guide each unique patient journey.^10^ Literature indicates that the essential components of a care pathway include: (1) the care plan; (2) pathway development and implementation by multidisciplinary teams (MDTs) (doctors, nurses, dieticians); and (3) its application to various facets of care (investigation, diagnosis, treatment).^11^ Mapping the South African GC pathway will influence healthcare practices in SA to enhance GC quality of care, increased patient safety and satisfaction, and optimal use of resources.^12^ Health policy may be influenced by leveraging the GC care pathway to inform national GC care guidelines for the unique South African ethnic and environmental diversity. These health policy changes may improve GC healthcare quality and patient outcomes.^13^

In SA, data regarding GC diagnosis and care are sparse; yet, GC is a dire condition with a substantial age standardised mortality rate of 0.31 per 100 000 people in males and 0.14 per 100 000 people in females.^14^ Further research is needed to understand the GC pathway considering the GC burden, incidence and mortality rates in SA.

The study aims to map the South African GC care pathway from diagnosis to management by HCPs involved in the GC patient journey, and to explore the barriers and facilitators to the effective flow of the GC care pathway.

## Research methods and design

### Study design

This research article follows a qualitative study design using in-depth HCP interviews.

### Setting

Participants in this study are HCPs from the public and private healthcare sectors in SA. The participants are HCPs who diagnose, treat or manage GC.

### Study population and sampling strategy

The interview participants included general practitioners (GPs), gastroenterologists (GIs), surgeons, oncologists, nurses, pathologists, dieticians and palliative care specialists in the private and public healthcare sectors from the three most populated provinces in South Africa – Gauteng, KwaZulu-Natal and Western Cape. The GP was the first point of contact and chain-referral (snowball) sampling was used to source other clinicians involved in the diagnosis, care and treatment of GC. The GPs were sourced by their likelihood of encountering cancer patients based on the size of their practice and geographical location. The interview guides were piloted by a GP, an oncologist and a surgeon to increase the accuracy and relevance of the questions for further dissemination to HCPs.

### Data collection

Primary data were collected from 30 HCPs using qualitative in-depth interviews via telephone call and online meeting platforms (Google Meet and Microsoft Teams), which were recorded for accurate transcription. The duration of the interviews ranged from 20 to 40 min per HCP over a 5-month period. Informed consent was received by all interview participants for participation and recording. There were eight sets of interview transcripts unique to the eight disciplines of HCPs. After the interviews were conducted, they were transcribed manually and precisely on Microsoft (MS) Word, followed by cleaning, formatting and processing of transcripts in MS Word. Files were cleaned to remove any duplicate text elements such as repeated questions, responses or words, to ensure accuracy and clarity of the transcriptions.

### Data analysis

Computer-assisted qualitative data analysis software (CAQDAS) MAXQDA was used to analyse the processed and formatted interview transcripts. A MAXQDA project was created to organise, analyse and visualise qualitative data efficiently, for efficient data management and comprehensive qualitative insights. MAXQDA streamlined the data analysis to maintain consistency and improve the rigour of the qualitative research.

Inductive qualitative analysis was employed to eliminate preconceived ideas and patterns. The inductively analysed transcript data allowed for the unbiased generation of concepts and themes. The first phase of the analysis for the generation of initial codes employed open coding in MAXQDA, which distinctly partitions the qualitative data to identify similarities and differences. Labels were assigned to different data segments that represent significant concepts. This step generated a large number of codes, displaying the thorough and distinct nature of the qualitative data. After open-phase coding in MAXQDA, similar codes were identified and merged to eliminate overlaps, ensuring each code represents only one concept for clarity of the data analysis.

After code finalisation, homogeneous codes were grouped together into sub-themes to classify the data for further analysis to obtain insights. Homogeneous sub-themes were further grouped into broader categories called themes, to capture overarching concepts of the data and to summarise the findings. Common patterns across the sub-themes were identified and organised into higher-level themes that represent the vital aspects of the data. After the analysis, a report was generated in MS Word.

### Ethical considerations

Ethical clearance to conduct this study was obtained from the University of the Witwatersrand Human Research Ethics Committee (Medical) (No. M220752).

## Results

The 8 disciplines of 30 HPCs comprising 5 GPs, 4 GIs, 4 surgeons, 5 oncologists, 2 pathologists, 5 nurses, 3 dieticians and 2 palliative care specialists, all had more than 10 years of experience treating cancer patients from both the public and private health sectors in SA. The HCPs comprised of an equal number of men and women distributed equally across the three most densely populated provinces — Gauteng, KwaZulu-Natal and Western Cape.

**FIGURE 1:**
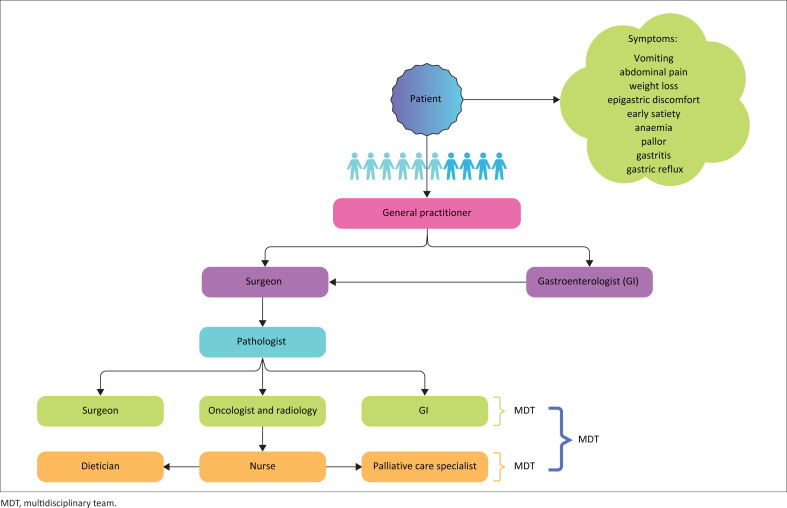
Gastric cancer care pathway in the South African private health sector.

**FIGURE 2:**
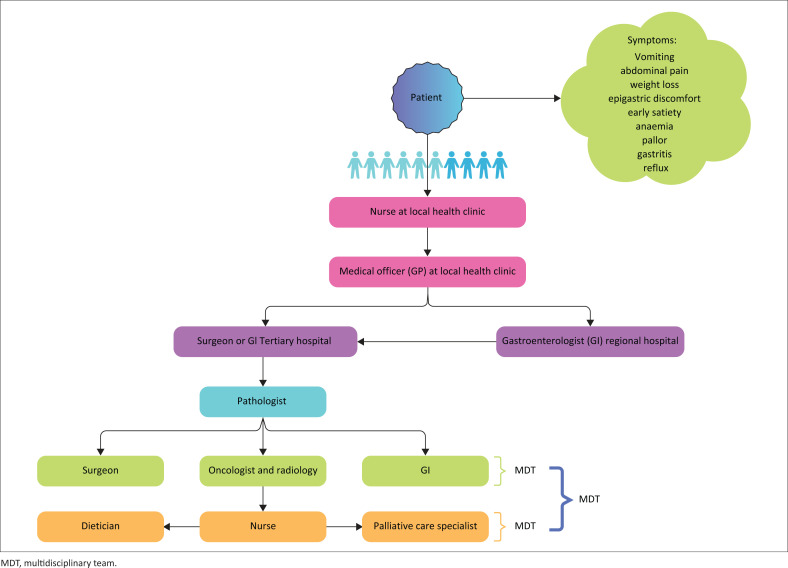
Gastric cancer care pathway in the South African public health sector.

The private and public healthcare sectors in SA follow a similar GC care pathway – with slightly more linearity in the public sector – regarding HCPs selection in the referral process and the diagnostic, staging and treatment regimens (Figure 1 and Figure 2). The generic flow of care from the initial consultation starts with the GP or nurse at a local public clinic followed by the GI, surgeon and pathologist. After pathology, the flow reverts to the surgeon and the introduction of the oncologist; and thereafter, the supportive functions by the nurse, dietician and palliative care specialist. The nurse is usually introduced into the pathway upon diagnosis of GC. The dietician and palliative care specialists are also introduced at the diagnosis step, but this differs between the private and public sectors. The stark differences between the two healthcare sectors are the time to diagnosis, staging of GC and the waiting periods for consultation with the various clinicians and GC treatment at each step.

The major themes that were identified in the qualitative analysis were:

- Referral and coordination processes in the GC care pathway include diagnostic processes, the role of HCPs in GC care pathway and MDT care.
- Public versus private sector healthcare system differences include disparities in diagnosis stage, disparities in treatment access and disparities in time and access to HCPs.
- Challenges and gaps experienced in the GC care pathway include delays in the GC diagnosis, Low GC index of suspicion by PCC and *H. pylori* detection.

Each of these three major themes are discussed in detail:

1. Referral and coordination processes in the GC care pathway describe the route from initial consultation with the PCC, which is a GP or nurse, to either a GI or surgeon for a gastric endoscopy depending on the symptoms such as gastric reflux, weight loss, nausea, dyspepsia, gastric disturbances, epigastric pain and anaemia that does not subside with treatment. The surgeon makes the GC diagnosis from the biopsy results provided by the pathologist. After a GC diagnosis, the oncologist is consulted and a treatment regimen is decided upon by the surgeon and oncologist based on the GC stage. The nurse is introduced to the care pathway to assist the oncologist and surgeon with treatment administration, side effect management, patient counselling and the patients’ bedside care. The dietician is consulted for optimal nutritional intake to prepare the patient for treatment and ensure optimal recovery after treatment. Palliative care is usually introduced to GC patients in late-stage diagnosis for improved comfort and quality of life. The analysis revealed a MDT approach for optimal care of GC patients. The MDT at the initial level comprises the GI, surgeon and oncologist, and the second level includes the nurse, dietician and palliative care specialist. The nurse is involved in both levels of the MDT.
2. Public versus private sector healthcare system differences: Healthcare system differences between the private and public sectors include GC staging disparities at diagnosis, endoscopy referral and scheduling, access to cancer specialists, timeliness and support for GC treatment, post-treatment management and follow-up in GC care. All the aspects of the public health sector are severely delayed compared to the private sector because of limited resources and inefficiencies in the public health sector.
3. Challenges and gaps experienced in the GC care pathway include *H. pylori* screening and detection in GC patients, and gaps in GC index of suspicion by PCC and GC diagnosis delays.

## Discussion

The aim of this study is to map the South African GC care pathway from diagnosis to various HCPs involved in the GC patient journey and to explore the barriers and facilitators to the effective flow of the GC care pathway. The GC care pathway indicates that the HCPs work in a MDT for optimal care of the GC patient in both the private and public health sectors. The findings from the study show that diagnostic process for GC involves the patient’s clinical examination, endoscopic biopsy testing and radiology. There are disparities in GC diagnosis, access to treatment and access to cancer specialists in both the private and public health sectors. These disparities are because of medical insurance differences in the private sector, and limited resources and staff inefficiencies in the public sector. There are significant delays to GC diagnosis, treatment and staging in the public sector compared to the private sector. *Helicobacter pylori* detection is a challenge in GC patients because of primary health practitioners not screening early enough and the absence of the bacteria in biopsy tissue. The Summary of themes, sub-themes, and quotes from healthcare professional interviews are indicated in Table 1.

**TABLE 1:** Summary of themes, sub-themes, and quotes from healthcare professional interviews.

Themes	Sub-themes	Quotations
Referral and coordination processes in GC care pathway	Diagnostic processes in GC care pathway	“If patient is over 40, losing weight, anaemic, not responding to proton pump inhibitors (PPI) then the patient is referred for a gastroscopy.” (participant 6, male, BI-Gastroenterologist) “GC is only confirmed with a scope. Biopsy of the lesion will be taken, and the confirmation will be made in the laboratory.” (participant 7, male, VGN-Gastroenterologist)
	Role of healthcare providers in GC care pathway	“Normally when the patient comes to the clinic, they would have already seen the surgeon, then they come in to see the oncologist and at that point they introduce the nurse as the chemo sister who will be doing the treatment plan. Nurse introduced at first consult after diagnosis.” (participant 22, female, AM-Nurse) “Epigastric pain or vomiting blood are symptoms that GC patients come in with, hence they are referred to surgeons for investigation into the abdominal pain or GI bleeding, and surgery and scoping can be done if needed on the investigation.” (participant 13, female, Dr LP-Surgeon) “Oncologist do not make diagnosis, the surgeon or GI specialist will diagnose the patient. Patients always referred to oncologist after diagnosis for treatment.” (participant 11, male, MN-Surgeon) “As palliative care nurse we are specialists and play a very active role, take history and assess patient. Diagnose pain, emotional state, spiritual state, psychosocial issues and refer to necessary people like social worker, spiritual healer.” (participant 26, female, Palliative Care)
	Multidisciplinary team care	“All members of the multidisciplinary team work well together and interact well with each other. All work together to provide good support to patient.” (participant 21, female, VR-Nurse) “HCPs in the multidisciplinary team are good with referring to the necessary healthcare professionals like the dietician or psychologist and have a good relationship with each other.” (participant 22, female, AM-Nurse) “Multidisciplinary team manage patient and all members consult with the patient. Surgeon and oncologist mostly manage the patient. GI will come in for further scopes if needed.” (participant 7, male, VGN-Gastroenterologist)
Public Vs. Private Sector Healthcare System	Disparities in diagnosis	“Within 24 hours in private sector for gastric carcinoma in lancet.” (participant 20, female, TG nurse) “In state it is highly variable and can possibly be a month to diagnose GC.” (participant 24, female, TP-Pathologist) “In state in patients where the HCP has a high index of suspicion then the scope can happen in 2 weeks provided the GI specialist is consulted and they understand the severity of the situation. If the GI is not consulted and an endoscopy is ordered, from first consult with the initial clinician to the endoscope can take 4 to 8 weeks.” (participant 7, male, VGN-Gastroenterologist)
	Disparities in treatment access	“Capacity at the hospital, office space, financial issues for transport to hospital. In the hospital, when patients have chemotherapy, it is difficult to navigate to the chemotherapy ward. The government hospital does not employ navigators.” (participant 15, female, SB oncologist) “The actual treatment not being available is very common and this is a delay as well. Office space for radiation is too small so they have a high backlog for radiation.” (participant 15, female, NV Oncologist) “Delays with surgery are due to scheduling of patients in the theatre and you have to share theatre space to other cancers and other conditions.” (participant 11, male, MN Surgeon)
	Disparities in time and access to Cancer Specialists	“When patients are referred to state and regional hospital, they are not thoroughly looked at, not examined, and not investigated properly. It is an absolute travesty in state hospitals. Referral letters are not read. The issues are with the doctors and nursing staff.” (participant 5, male, PDR GP) “Huge discrepancy between private sector and state sector. In state, you must wait a long time for a scope, the patient has to wait to come back to the clinic for histology, then the referral to surgeon and there is a delay there and a further delay to see oncologist. All these delays cause the gastric cancer to spread before being treated.” (participant 6, male, BI-Gastroenterologist) “In state sector patients on average wait 6 months from one HCP consult to the next. There is no continuity in state and no commitment to the patient in state. The patient is just a number in state.” (participant 17, female, DP oncologist)
Challenges and gaps experienced in the GC care pathway	Delays in the diagnosis, staging and treatment of GC	“in state we very rarely see gastric cancer patients with early disease, most patients – 80% of patients - are in state are stage 3 or 4, and they are terminal and treatment will only improve survival.” (participant 15, female, NV Oncologist) “In private sector there is no delay” (participant 14, male, JR Oncologist) “In state 20% are early stage. Locally advanced (stage 2 and 3) mostly about 50 – 60% of patients. Stage 4 cases are about 30 to 40%.” (participant 16, female, GD Oncologist) “in private Only in advanced setting there is delay, in early in neo adjuvant and adjuvant setting there is no delay. In metastatic setting or locally advanced irresectable there may be delay in funding of trastuzumab if patient is HER2+.” (participant 16, female, GD Oncologist) “in state it is late presentation 80%, early 20%, in private it is 50 -50 with early and late stage” (participant 15, female, NV Oncologist)
	*H. pylori* detection	“Stool and breath test sensitivity and specificity may not be accurate and may not distinguish between current and previous infection.” (participant 25, female, KF pathologist) “Does not know why clinicians order or do not order H. pylori testing but in the laboratory the specimen is always tested for H. pylori.” (participant 25, female, KF pathologist) “Pathologists will always look at biopsy samples for H. pylori. Chance of H. pylori in the tumour is rare because H. pylori does not like abnormal mucosa that result from malignancy, they like normal gastric mucosa.” (participant 26, female, TP pathologist) “If there is a large ulcerating lesion, then biopsy will be taken but H. pylori will not show cos H. pylori is not in the tumour itself but in other parts of the stomach, that’s why it is missed. Cannot do biopsy in normal stomach at same time as tumour biopsy because the same forceps are used and the tumour cells may spread to healthy parts of the stomach.” (Participant 9, male, EL Gastroenterologist)

GC, Gastric cancer; GI, Gastroenterologist; HCP, Healthcare professional.

The GC diagnostic process revealed in this study are aligned with literature where recently diagnosed GC patients presents with an epigastric endoscopy report for mild symptoms, including dyspepsia and reflux, but also for signs and symptoms indicative of advanced disease, such as gastrointestinal bleeding, anaemia, dysphagia, weight loss and severe vomiting. Gastric endoscopy with a computed tomography (CT) scan and endoscopic ultrasound provides a GC diagnosis and clinical staging for GC. Positron emission tomography (PET) scans are required to detect advanced GC disease and peritoneal washing.^15^

The 2022 National Comprehensive Cancer Network (NCCN) and the 2022 European Society for Medical Oncology (ESMO) guidelines recommend a MDT approach for GC treatment and patient care which supports the findings of this study.^16,17^ The guidelines state that GC patient management requires a few disciplines comprising surgical oncology, medical oncology, radiation oncology, GI, radiology and pathology.^16,17^ Findings from this study reveals that the GC patient management in SA follows a similar structure comprising of the surgeon, clinical oncologist (includes medical and radiation oncology), GI, radiology and pathology. The guidelines mention the importance of supportive care provided by the nurses, palliative care specialist, nutritionists and social workers which aligns with the findings of this study where the nurses, dieticians, palliative care specialists and GPs comprise the supportive MDT health workers.^16,17^This study found that nurses form part of the primary MDT as well as the supportive MDT. Literature supports this finding as nurses are known as oncology ‘coordinators of care’ or ‘navigators’ and are integral in relaying physical and psychosocial patient information to the specialists in the MDT and administering specialist prescribed treatment to patients.^18^ Multidisciplinary team care for GC patients have been shown to reduce time to treatment, unnecessary staging tests and treatment inconsistencies.^19^ These factors improve quality of care, staging techniques like endoscopic ultrasounds and consistent patient management.^19^

A strong theme that emerged was the disparities in the private healthcare system compared to the public healthcare system especially with regards to diagnosis, treatment and access to cancer specialists. In SA, 84.2% and 15.8% of the population uses public health services and private medical insurance, respectively.^20^ Majority of the population are dependent on the under-resourced public health sector and the wealthier minority use private healthcare services which are well resourced with a system that works seamlessly for both patients and HCPs.^4^ This study found that GC diagnosis is delayed in both the private and public sector, but greater delays exist in the public health sector. Research shows that majority of patients using public health services have poor health seeking behaviour and financial constraints that limit them from accessing healthcare clinics and hospitals.^21,22^ Private healthcare patients live closer to hospitals, have means to travel and funds to pay for diagnostic testing.^22^ Herein lies a plausible explanation for a greater proportion of public health users experiencing longer delays in their GC diagnosis as well as being diagnosed at a more advanced GC stage than private healthcare users.

In the private sector, there are generally no delays to GC treatment and HCP access because of the patients being members of private medical insurance schemes which covers patient care at private hospitals.^23^ If patients experience delays, they are because of instances where the patient may need to pay a co-payment for their diagnostic endoscopy if their medical insurance plan does not cover the entire cost or if their medical insurance plan only covers the prescribed minimum benefit for GC, which is the equivalent of public sector treatment. In this case, the patient must pay for any additional targeted therapy or immunotherapy for metastatic GC.^23^ If the medical insurer approves additional specialised treatment, there is usually a waiting period of 5 to 30 working days for treatment approval. Targeted therapy and immunotherapy are not available in public sector hospitals where only surgery, chemotherapy and radiation are available.^1^

Research on public health services in SA shows poor quality of care and poor patient outcomes owing mainly to ineffective leadership at hospitals and clinics.^24^ Public healthcare funds and budget are misused, and the clinics and hospitals have old and often broken infrastructure that are unable to accommodate the large number of patients.^6^ Medicinal and staff shortages are a challenge in the South African public health system, and this issue is exacerbated by reduced staff capacity and competencies.^25,26^ The shortage of medicines and medical staff, suboptimal infrastructure and staff competencies, and poor safety and working conditions in the public health^7,11^ sector align with the delayed time to GC diagnosis, delayed treatment access, limited treatment options, and restricted nutritional and palliative care identified in this study.

The Barriers and facilitators to the effective flow of the GC care pathway are indicated in Table 2. Challenges and gaps identified in the GC care pathway exist in both the private and public health sectors and include a low index of suspicion for GC by the PCC, *H. pylori* screening delays and poor public health service. Early recognition and diagnosis of GC for timely treatment requires accessible, good quality health services coupled with optimal healthcare workers and resources.^27^ This is lacking in the South African public health sector which is responsible for the care of 84% of the population. Herein lies the low index of suspicion from PCC for GC which leads to delayed referral for diagnosis and limited treatment options. The implications of delayed diagnosis and limited treatment options range from increased anxiety of the patients and caregivers, presentation of advanced disease stage requiring multi-modality treatment, and poor prognosis and survival rates.^28,29^

**TABLE 2:** Barriers and facilitators to the effective flow of the gastric cancer care pathway.

Barriers to an effective GC care pathway	Facilitators to an effective GC care pathway
Public healthcare sector	Private healthcare sector
Large patient numbers	Small patient numbers
Delayed time to diagnosis	Patients living closer to hospitals
Delayed time to treatment	Timely diagnosis
Poor health-seeking behaviour	Health awareness from patients
Low index of suspicion at primary care level	High index of suspicion at primary care level
Medical insurance waiting period for treatment approval	Medical insurance covering all treatment in the guidelines
Treatment shortage	Multidisciplinary team care
Staff shortage	
***H. pylori*** screening delays	
Community bias against chemotherapy	
Poor patient referral to specialists	

GC, gastric cancer.

Literature indicates that the leading risk factor for peptic ulcer disease and GC is *H. pylori* infection with a prevalence of over 80% in adults from developing countries.^30^ Recent data indicate 89% of the global GC prevalence may be attributed to *H. pylori* infection.^30^ This information differs to the findings of this study where pathologists reported that *H. pylori* was not present in the GC biopsy tissue. Surgeons biopsy potentially malignant tissue for GC testing and this tissue does not test positive for *H. pylori* as the bacteria travels away from malignant tissue to areas of the GI tract with healthy gut mucosa.^31^ This is a challenge because GC in SA cannot be attributed to *H. pylori* infection because of *H. pylori* not being screened at the early stage of only peptic ulcer disease, and screening from malignant GC biopsy tissue is too late for *H. pylori* presence.

### Limitations, strengths and recommendations

The limitation of this study notes the exclusion of patient participation and the lack of verification of the pathway from the patient perspective. Barriers to the GC care pathway at a patient level was not identifiable because of exclusion of patient participation. The difference between the oncology and radiology practice structure between the Gauteng province and the rest of the SA is a limitation as the medical oncologist from Gauteng cannot practice radiology and thus cannot comment on this treatment modality. The reflexivity limitation of the primary investigator and interviewer was that the Western Cape and Gauteng provinces had superior treatment and care for GC patients because of the investigator living in KwaZulu-Natal and witnessing poor healthcare from the KwaZulu-Natal public sector hospitals. This bias was cleared up in the interview stage as participants reported similar insights from all three provinces.

The strengths of this study include it being the first study to explore the GC care pathway in SA, the participation of eight different health worker disciplines, HCP participation from the three major provinces in SA, HCP participation from both the private and public health sectors. Insights gathered on other areas of GC treatment and care like nutrition, targeted therapy and staging of GC are also notable strengths.

Future research is necessary to address the gaps and limitations of this study. There is a need for further studies on *H. pylori* by collaborating with GPs and pathologists to explore the causal relationship between *H. pylori* and GC in SA.^32^ A study on the GC care pathway from a patients’ perspective will allow for verification of this study’s findings and an understanding of the patients’ GC treatment and management journey. Research into the tolerability and efficacy of the available treatment and the subsequent quality of life of patients in the public and private health sectors will provide further insight into the outcomes of current practices in the different health sectors.

Recommendations from this research include the training of PCC by specialist HCPs to increase their GC index of suspicion,^32^ *H. pylori* screening earlier in the GC patient journey to decrease time to diagnosis and time to treatment.^31^ Exploring possible synergistic agreements between the private and public health sectors to expedite GC diagnosis in the public health setting by leveraging the high functioning private healthcare system.^21,22^ The robust cohort of study participants and their experience with treating GC have provided valuable insights, which can aid in shaping practice of MDTs for GC and harmonise treatment and management decisions at all stages in the GC patient journey.^33^ An advisory board for a standardised approach to GC care in the private and public sector with a similar robust HCP cohort can influence GC healthcare policies at a national level.^33^

## Conclusion

In conclusion, this study’s findings suggest that thorough initial staging upon GC diagnosis provides a basis for a vigorous treatment plan, enhanced decision making on surgery and treatment administration. These steps will facilitate the effective flow of the GC care pathway and provide patients with a solid understanding of their disease and prognosis. An effective care pathway may assist in identifying a potential for cure or increased quality of life as early as possible in the patients’ treatment plan.^15^ Guidelines encourage MDT decision-making to reduce and relieve suffering of GC patients and improve their quality of life irrespective of their GC stage by providing optimal treatment options. A MDT approach for optimal treatment and patient care is believed to be the best method for prolonged life.^16^ A South African national consensus for GC care via a MDT, with emphasis on early detection and diagnosis to aid in a robust treatment plan for improved patient outcomes is warranted. A national consensus will aid in public health strategies for a uniform and patient-specific approach for GC care in SA.
